# Mouse Syngeneic Melanoma Model with Human Epidermal Growth Factor Receptor Expression

**DOI:** 10.3390/pharmaceutics14112448

**Published:** 2022-11-12

**Authors:** Tatiana A. Slastnikova, Andrey A. Rosenkranz, Alexey V. Ulasov, Yuri V. Khramtsov, Tatiana N. Lupanova, Georgii P. Georgiev, Alexander S. Sobolev

**Affiliations:** 1 Laboratory of Molecular Genetics of Intracellular Transport, Institute of Gene Biology, Russian Academy of Sciences, Moscow 119334, Russia; 2 Department of Biophysics, Faculty of Biology, Lomonosov Moscow State University, Moscow 119991, Russia

**Keywords:** human epidermal growth factor receptor, syngeneic mouse melanoma, Cloudman S91 (M3), preserved in vivo expression

## Abstract

The development of epidermal growth factor receptor (EGFR)-targeting agents for the treatment of malignant melanoma requires cheap and easy animal tumor models for high-throughput in vivo screening. Thus, the aim of this study was to develop mouse syngeneic melanoma model that expresses human EGFR. Cloudman S91 clone M3 mouse melanoma cells were transduced with lentiviral particles carrying the human EGFR gene followed by a multistep selection process. The resulting M3-EGFR has been tested for EGFR expression and functionality in vitro and in vivo. Radioligand assay confirmed the presence of 13,900 ± 1500 EGF binding sites per cell at a dissociation constant of 5.3 ± 1.4 nM. M3-EGFR demonstrated the ability to bind and internalize specifically and provide the anticipated intracellular nuclear import of three different EGFR-targeted modular nanotransporters designed for specific anti-cancer drug delivery. Introduction of the human EGFR gene did not alter the tumorigenicity of the offspring M3-EGFR cells in host immunocompetent DBA/2J mice. Preservation of the expression of EGFR in vivo was confirmed by immunohistochemistry. To sum up, we successfully developed the first mouse syngeneic melanoma model with preserved in vivo expression of human EGFR.

## 1. Introduction

Despite significant progress in clinical oncology, cancer still remains one of the leading causes of death worldwide [1]. Thus, more and more research is being conducted in the anticancer drug development field. Among promising anti-cancer therapeutics, those aimed at the epidermal growth factor receptor (EGFR) family are being extensively developed [2]. EGFR represents a 170 kDa transmembrane glycoprotein which, upon activation, switches on downstream signaling pathways responsible for cell survival, differentiation and proliferation [3]. Consequently, EGFR dysregulation, overexpression or aberrant functioning caused by various mutations or gene amplification are typical for many human solid cancers and are usually associated with an adverse prognosis [4,5]. EGFR overexpression has been detected in a significant proportion of head and neck, cervical, non-small cell lung, bladder, pancreatic and colon cancers, as well as in mesothelioma, glioma and other cancers [6], making EGFR a very attractive target for various anti-cancer agents in recent decades.

Numerous EGFR-targeting agents have already been and are being developed. Among them, drug delivery vehicles using EGFR to enter the cancer cells are of specific interest, as various cytotoxic agents can be delivered this way to EGFR overexpressing cells. Modular nanotransporters (MNTs), belonging to this class of delivery systems, represent a promising tool for delivery of anticancer drugs into the nuclei of cancer cells with increased expression of internalizable surface receptors [7,8]. The efficacy of EGFR-targeted MNT bearing either photosensitizers or Auger electron emitters for the treatment of different model tumors, including epidermoid [9] and bladder carcinomas [10] has demonstrated in vitro and in vivo.

In recent years, interest has increased in the use of EGFR-targeting agents to treat malignant melanoma, which is responsible for most deaths caused by skin cancers. EGFR overexpression is rather frequent in melanoma, mainly in its most deadly nodular and uveal subtypes. Known for its ability to modulate tumor invasiveness, EGFR expression was demonstrated to correlate positively with poor survival and increased rates of metastasis in melanoma patients [11]. Despite the excellent long-term treatment benefit of BRAF/MEK inhibitors, acquired resistance to this treatment requires the search for new molecular targets involved in this process. As EGFR activation plays a significant role in the development of resistance to anticancer drugs, including BRAF inhibitors [12], EGFR is regarded as a very attractive candidate.

Each drug development study requires routine and extensive use of in vitro and in vivo models to screen numerous newly created anticancer agents for selection of the most potent ones, which will enter more expensive preclinical and further clinical trials. There are numerous EGFR-expressing human cancer cell lines, including melanoma ones which are successfully used for in vitro screening of the EGFR-targeted agents being developed. Though a number of different more or less sophisticated in vivo tumor models now exist [13], syngeneic mouse tumor models still remain the cheapest, easiest and most reproducible ones for the very first in vivo screening of anti-cancer agents. Limited availability of EGFR strongly positive mouse tumorigenic cancer lines (and no such melanoma cells) hinders the use of this convenient variant for the next-step preliminary in vivo testing via in vitro screening EGFR-targeted agents. Moreover, though mouse and human EGFR sequences are quite conservative, they do differ, so that some EGFR-targeted agents possess specificity only to human EGFR [14], requiring the use of a tumor model expressing human EGFR. Published attempts to develop an EGFR-expressing mouse melanoma model resulted in tumors, which, unfortunately, lost their EGFR expression in vivo in immunocompetent animals [15,16].

Thus, the aim of this work was to create a mouse syngeneic melanoma model that expresses a significant level of human EGFR.

## 2. Materials and Methods

### 2.1. Cell Lines

A431 human epidermoid carcinoma and Cloudman S91 mouse melanoma (clone M3) cells were purchased from ATCC. Cells were cultivated in either Dulbecco’s Modified Eagle Medium (DMEM) (A431) or DMEM/F12 medium (M3 and M3-EGFR) supplemented with 10% fetal calf serum, 50 µg/mL gentamicin. Cell lines were maintained at 37 °C in a 5% CO2 humidified atmosphere.

### 2.2. Modular Nanotransporters (MNTs)

**Figure 1:**
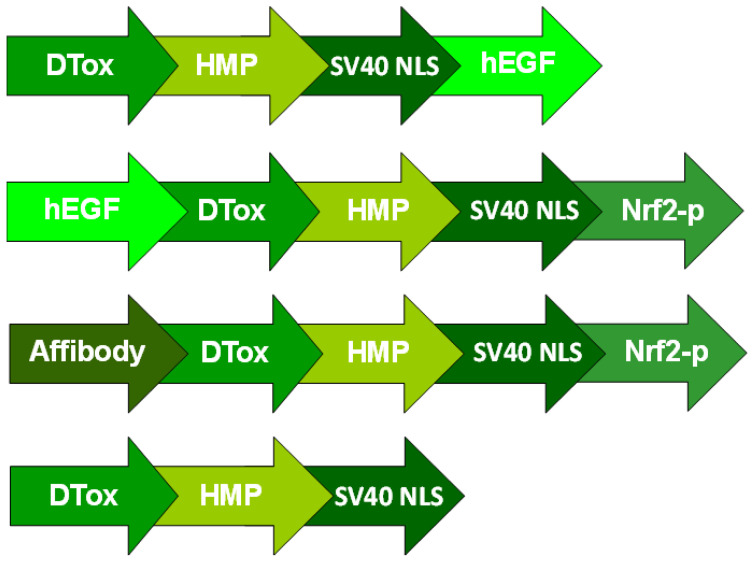
Schematic presentation of different MNTs used in this study. DTox—translocation domain of diphtheria toxin; HMP—*E. coli* hemoglobin like protein (carrier module), SV40 NLS—an optimized SV-40 nuclear localization sequence; hEGF—human epidermal growth factor, Affibody—antibody-like EGFR affibody polypeptide, Nrf2-p—functional polypeptide sequence from nuclear factor erythroid 2-related factor 2 (Nrf2) for interaction with intracellular proteins.

The following MNTs were used in this work: DTox-HMP-NLS-EGF (MNT-EGF), EGF-DTox-HMP-NLS-[Nrf2-peptide] (EGF-MNT), [anti-EGFR-affibody]-DTox-HMP-NLS-[Nrf2-peptide] (affibody]-MNT), DTox-HMP-NLS (non-targeted MNT), where DTox is a translocation domain of diphtheria toxin (endosomolytic module), HMP is *E. coli* hemoglobin like protein (carrier module), NLS is an optimized SV-40 nuclear localization sequence (nuclear pore import module), EGF, epidermal growth factor (ligand module), affibody, antibody-like EGFR affibody polypeptide (ligand module) [17], Nrf2-peptide served a functional polypeptide sequence for interaction with intracellular proteins. These MNTs were produced and purified as described previously [17,18]. The schematic presentation of the employed MNTs structure is depicted in Figure 1.

### 2.3. Conjugation of Alexa Fluor 647 to Modular Nanotransporter (MNT)

A freshly prepared solution of Alexa Fluor 647 succinimidyl ester (Molecular Probes, Eugene, OR, USA) (2 mg/mL) was added to the MNT EGF-DTox-NLS-HMP solution in carbonate buffer, pH 8.6 in molar excess of 5:1. The reaction mixture was incubated overnight at +4 °C with gentle mixing. The resulting MNT-Alexa647 was purified from unreacted fluorophore and by-products by 5 cycles of ultra-filtration via Amicon Ultracel-30K.

### 2.4. Conjugation of Sulfo-Cy3 to Cetuximab

Cetuximab (trade mark—Erbitux, Merck, Darmstadt, Germany) was dialyzed against phosphate buffer saline, pH 8.0. A freshly prepared solution of sulfo-Cy3 succinimidyl ester (Lumiprobe, Moscow, Russia) was added to the cetuximab (5 mg/mL) solution in carbonate buffer, pH 8.6 in molar excess of 4:1. The reaction mixture was incubated for 3 h at room temperature with gentle mixing. The resulting cetuximab-Cy3 was purified from unreacted fluorophore and by-products by size-exclusion chromatography through a PD-10 column (GE Healthcare Life Science, Little Chalfont, UK) in phosphate-buffered saline (pH 7.5).

### 2.5. Labeling of Epidermal Growth Factor with ^125^I

Labeling of human EGF (Sigma Chemical, St. Louis, MO, USA) was performed with ^125^I (Khlopin Radium Institute, Russia) using Iodogen (1,3,4,6-tetrachloro-3α,6α-diphenylglucoluril, Sigma, USA). For the reaction, 10 µg of the EGF solution (1 mg/mL) and 21 MBq of ^125^I in 0.05 M sodium borate buffer (pH 8.5) were incubated for 15 min at room temperature in glass vials coated with 10 µg of Iodogen with continuous stirring. After the reaction, tyrosine was added into the reaction mixture up to a final concentration of 2.5 mM. The labeled EGF was purified by size-exclusion chromatography through a PD-10 column (GE Healthcare Life Science, UK) in phosphate-buffered saline (pH 7.5). The initial specific activity of the labeled protein was 12 TBq/mmol of EGF.

### 2.6. Development and Selection of M3 Cells with Expressing Functional EGFR

M3 cells were transduced with lentiviral particles carrying the human EGFR (EGFR) gene. Cell transduction was performed by Evrogen (Moscow, Russia). The human EGFR gene from plasmid DNA containing the EGFR gene (Addgene #32751) has been cloned into lentiviral vector pLVT (Evrogen, Moscow, Russia). The sequence of the primers used to subclone EGFR into pVLT were:

EGFR-Nhe: 5′-GCTAGCCAGCGATGCGACCCTC-3′

EGFR-Sall: 5′-GTCGACTGCTCCAATAAATTCACTGCTTTG-3′

Thus, a lentiviral pLVT-EGFR vector encoding EGFR under the control of the alpha-human elongation factor 1 subunit (EF1a) was developed.

The resulting lentiviral vector was used for production of lentiviral particles in an HEK293T cell culture. HEK293T were transfected with a mixture of lentiviral vector DNA and helper plasmids pRSV-REV, pCMV-VSV-G, and pCMV-GAG encoding structural proteins of viral particles and vesicular stomatitis virus envelope glycoprotein, using FuGene6 transfection reagent (Promega, Madison, WI, USA) according to the manufacturer’s protocol. A culture medium containing viral particles was collected 48–50 h after transfection, clarified by low-speed centrifugation and filtered through a 0.45 µM Millipore filter. The presence of lentiviral particles in the culture liquid was confirmed by measuring p24 capsid protein concentration using the “HIV-1 p24-antigen-EIA-BEST” kit (Vector-Best, Novosibirsk, Russia). M3 cells were transduced with the obtained LVT-EGFR lentiviral particles at multiplicity of infection of either 50 or 150 TU per cell. After a week of cultivation, transduced cells were frozen and provided to our research group by Evrogen (Russia).

**Figure 2:**
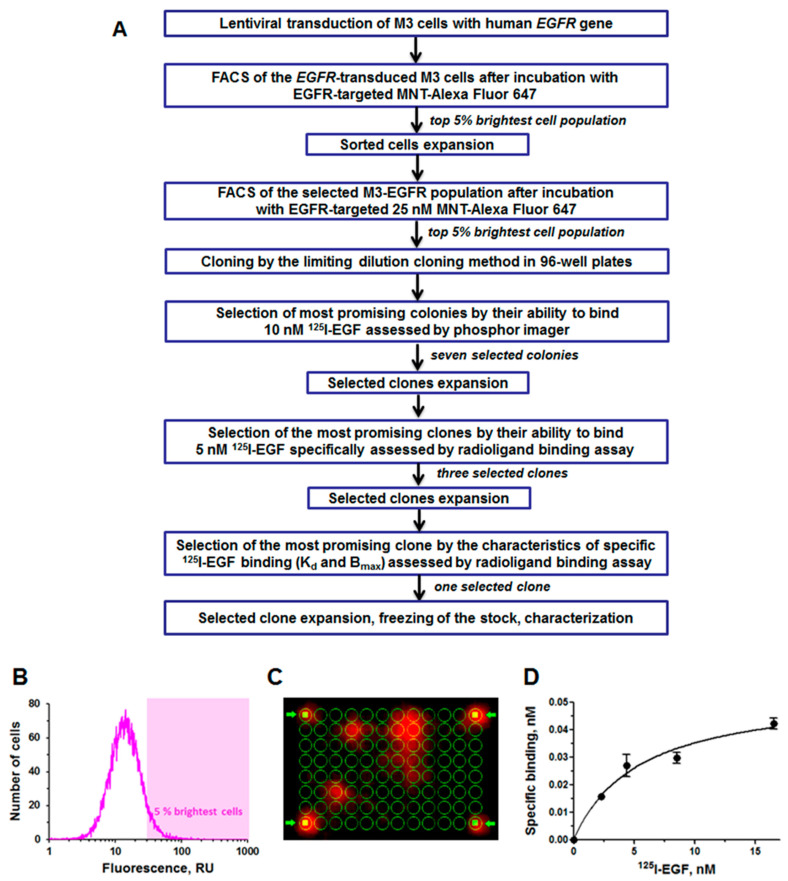
Development and selection of M3 cells with expressing functional epidermal growth factor receptor (EGFR). (**A**) Flow-chart illustrating the selection process step-by-step, (**B**) Fluorescence-activated cell sorting of initial M3-EGFR cells incubated with EGFR-targeted modular nanotransporter labeled with Alexa Fluor 647, the top 5% brightest cells that were sorted and used for further subcloning are highlighted. (**C**) Radioactivity distribution obtained by Storm 865 phosphorimager on the storage phosphor screen incubated with 96-well plate with M3-EGFR colonies after 24 h incubation of this 96-well plate with 10 nM ^125^I-EGF. The four bright marks pointed by arrows at the corners of the screen were made manually by applying radioactivity into the corner wells of the plate for adjustment 96-well plate green mask. (**D**) Radioligand ^125^I-EGF binding to finally selected M3-EGFR clone.

To obtain a Cloudman melanoma line expressing functional human EGFR, this polyclonal population of cells transduced with pLVT-EGFR was sorted using an Epics Altra flow cytometer-sorter (Beckman Coulter, Brea, CA, USA). To do this, EGFR-targeted MNT EGF-DTox-NLS-HMP labeled with Alexa Fluor 647 (see Section 2.3) was used. Cells transduced with pLVT-EGFR were seeded into 24-well plates, incubated with 25 nM MNT-Alexa647 for 48 h, detached from the wells with 0.25% Trypsin in 0.02% Versene in a balanced Dulbecco’s salt solution (Paneco, Moscow, Russia), centrifuged, resuspended in a Versene solution with 0.5% serum and finally sorted. To obtain a cell population enriched with cells expressing the highest level of functional EGFR, the top 5% of cells with the highest fluorescence were selected. The selected cells were expanded, and the sorting procedure was repeated one more time. These double-selected cells were cloned by the limiting dilution cloning method in 96-well plates, followed by subsequent 24-h incubation with 10 nM ^125^I-EGF. Afterwards, the cells were washed twice, a fresh medium was added to the wells and the plates were placed overnight on a storage phosphor screen in a CO_2_ incubator. Then, the distribution of radioactivity in the wells of the plates was determined using a Storm 865 phosphorimager (GE Healthcare, Danderyd, Sweden). As a result, seven M3-EGFR clones with the highest ^125^I-EGF binding ability were selected. These clones were grown in culture flasks and plated on 24-well plates to test for specific binding to ^125^I-EGF. Based on the results of a spot (one ^125^I-EGF concentration point) test for ^125^I-EGF binding, the three most promising clones were selected, for which standard EGF binding assay (see Section 2.7) has been performed. The clone that showed significant specific binding to EGF was grown and once again subjected to the fluorescence-activated cell sorting procedure described above. The flow-chart illustrating the selection process step-by-step is depicted in Figure 2.

### 2.7. EGF Binding Assay

^125^I-iodoEGF was used for binding assay in 24-well plates. Serial dilutions of ^125^I-iodoEGF in the sodium bicarbonate-deprived medium supplemented with 10 mg/mL bovine serum albumin and 20 mM 4-(2-hydroxyethyl)-1-piperazineethanesulfonic acid (HEPES, pH 7.5) were added into the wells, and the cells were incubated with ligands for 18 h at 4 °C. The addition of 1 μM nonlabeled EGF was used to measure nonspecific binding. The cells were washed three times with the same ice-cold medium on ice, lysed in 1 M NaOH for 30 min, and the radioactivity associated with the cell lysates was measured in γ-counter RiaGamma 1271 (LKB, Luleå, Sweden). The number of receptors and the EGF affinity constant for the receptor were determined using the GraphPad Prism 6 software package (GraphPad Software Inc., La Jolla, CA, USA) from the equation describing the reversible equilibrium interaction of a monovalent ligand with a monovalent receptor.

### 2.8. EGFR Expression in M3-EGFR In Vitro

To study EGFR expression, cells were grown on coverslips, washed with Hank’s solution, and fixed with cold methanol for 10 min. Inhibition of endogenous peroxidases was performed by incubation in a solution of 3% hydrogen peroxide in phosphate buffer saline (PBS), pH 7.4 for 7 min, followed by PBS wash for 5 min. Non-specific binding was blocked with 5% dry nonfat milk and 1% bovine serum albumin in PBS for 30 min. Then, the cells were incubated with rabbit anti-EGFR antibodies (catalog # ab 47439, Abcam, Waltham, MA, USA) diluted 1:40 for 12 h. Then, the cells were washed 3 times for 5 min with PBS pH 7.4, incubated with Rabbit-specific HRP conjugate (Goat Anti-Rabbit IgG H&L (HRP), catalog # ab97051, Abcam, USA) for 20 min, washed 4 times with PBS for 5 min, followed by incubation with a horseradish peroxidase substrate (DAB Plus substrate, Abcam), followed by two 5-min washes with PBS and two 5-min washes with deionized water. The nuclei were stained with Mayer’s hematoxylin for 2.5 min. After staining, serial washing with water, 50%, 70%, and 96% ethanol solutions (3 min each) was performed. For final drying, the preparations were treated with orthoxylene and then embedded in a polyvinyl alcohol-based embedding medium (Mowiol medium, Sigma-Aldrich, St. Louis, MO, USA). Stained cells were imaged using an Apotome 2 Imaging microscope (Zeiss, Jena, Germany) with a 40× objective, NA = 0.75. Semi-quantitative analysis of the resulting images was performed using ImageJ Fiji software ((National Institutes of Health, Bethesda, Rockville, MD, USA.) following the published protocol [19,20].

### 2.9. Study of EGFR-Targeted MNTs Endocytosis in M3-EGFR Cells

M3-EGFR cells were grown on coverslips placed in the wells of a 24-well plate for 24 h in a DMEM/F12 medium supplemented with 10% FBS. Then, the medium was changed to the fresh one supplemented with 300 nM of either one of EGFR-targeted MNTs (MNT-EGF, EGF-MNT, affibody-MNT) or control non-targeted MNT. After 2 h incubation, the cells were washed 3 times with Hanks′ Balanced Salt solution (Paneco, Russia), fixed with cold methanol for 10 min, and stored at −40 °C. Then, the fixed M3-EGFR cells were incubated with a blocking solution for 30 min to block non-specific binding, washed with PBS pH 7.4, and receptors were stained with mouse primary antibodies labeled with fluorescent dye Alexa Fluor 647 anti-His-Tag antibodies (Penta·His Alexa Fluor 647 Conjugate, catalog # 35370, Qiagen, Hilden, Germany, dilution 1:500). The preparations were incubated with antibodies for 12 h in the dark. After the incubation time, the cells were washed with PBS again, incubated for 1 h with secondary antibodies (Rabbit Anti-Mouse IgG H&L (Alexa Fluor^®^ 647) preadsorbed, catalog # ab 150127, Abcam, UK, dilution 1:400), washed and incubated for 1 h with 4’,6-diamidino-2 -phenylindole (DAPI, ThermoFisher, Waltham, MA, USA) for nuclear staining. The slides were embedded in a Mowiol medium, cells were imaged by confocal laser scanning microscopy using an LSM-510 META NLO microscope (Carl Zeiss, Jena, Germany) with a 63× objective, NA = 1.4. DAPI fluorescence was detected using a two-photon excitation with a pulsed femtosecond laser at a wavelength of 760 nm and registration in 397–495 nm window. Alexa-647 fluorescence excitation was performed with a laser with a wavelength of 633 nm, and fluorescence was recorded in 645–720 nm window using one Airy aperture. To quantify the ability of MNTs to enter the nuclei of M3-EGFR cells, the nuclear fluorescence in the Alexa Fluor 647 channel was analyzed using the built-in software of a confocal microscope. Nuclear fluorescence was assessed by isolating a circular area, in which the image of the nucleus was placed in the DAPI fluorescence channel. The average fluorescence intensity in the Alexa647 fluorescence channel was calculated over the entire region. To eliminate the image background fluorescence, the same areas were isolated within the background part of the image, after which the average background fluorescence was measured and subtracted from the fluorescence of the nuclei. Then, the average fluorescence intensity of the nuclei (n = 37–118) was obtained.

### 2.10. Flow Cytometry Studies of Cetuximab-Cy3 Interaction with M3-EGFR Cells

To study human EGFR expression, the cells (M3-EGFR, M3 and A431) were seeded in 12-well plates. Two days later, the medium was refreshed with the fresh one, containing 20 nM cetuximab-Cy3, followed by 2-h incubation at 37 °C in a 5% CO_2_ humidified atmosphere. Then, the cells were washed and detached using a Versene solution, containing 20 nM cetuximab-Cy3, followed by centrifugation at 300 rpm for 5 min. The cell pellets were resuspended in the ice-cold Versene solution and immediately analyzed by flow cytometry using MACSQuant Analyzer VYB (Miltenyi Biotec, San Diego, CA, USA). A total of 1.9–6.6 × 10^5^ gated events were collected per sample.

To measure nonspecific interaction, parallel wells with 20 nM cetuximab-Cy3 in the presence of 1 µM non-labeled cetuximab and 1 µM of human EGF were processed in the same way. To assess cell autofluorescence, the addition of cetuximab-Cy3 was omitted.

### 2.11. Animal Studies

The experimental protocol was approved by the Institute Commission for Animals. M3 and M3-EGFR tumors were established in DBA/2 mice (n = 19 for M3 and n = 7 for M3-EGFR) by subcutaneous injection of 2 million cells suspended in a 40 μL serum-free medium into the back flank region. When the tumors reached 5–10 mm in diameter, the mice were euthanized and the tumors were taken, cut into 2–3 mm pieces, embedded into PolyFreeze Tissue Freezing Medium SHH0026 (Sigma) and snap-frozen in liquid nitrogen vapor. Then, they were cut into 10 µm sections using CM1510 cryotome (Leica, Wetzlar, Germany) at −20 °C. Immediately after placing them on a glass slide, sections were fixed in with an acetone-methanol mixture (2:3) for 10 min at −20 °C. After fixation, the sections were air-dried at room temperature and stored in a refrigerator until use. Immunohistochemistry staining to detect EGFR was performed the same way as with the fixed cells (see Section 2.8).

### 2.12. Statistics

The data were analyzed using GraphPad Prism 6 software (GraphPad Software Inc., San Diego, CA, USA). One-way analysis of variance (ANOVA) with Tukey’s multiple comparisons test was carried out to test for significant differences between the means, unless otherwise stated.

## 3. Results

### 3.1. EGF Binding to M3-EGFR Cells

After the double fluorescence-activated cell sorting procedure followed by ^125^I-EGF binding assay selection, only one of the M3-EGFR clones showed significant specific binding to ^125^I-EGF (Figure 2D) with an affinity constant of K_d_ = 5.3 ± 1.4 nM, which is typical for the ^125^I-EGF to EGFR binding equilibrium dissociation constant (K_d_) [10]. The assessed number of receptors was 13,900 ± 1500 per cell, which lies within the typical range (8 × 10^3^–3 × 10^5^ receptors per cell) of EGFR expression in human melanomas [21,22,23,24]. A further fluorescence-activated cell sorting process of this clone (2b4) resulted in three daughter M3-EGFR clones with similar EGF binding characteristics. Based on this, the selection process was completed and 2b4 clone was used for further experiments.

### 3.2. EGFR Expression in M3-EGFR In Vitro

**Figure 3:**
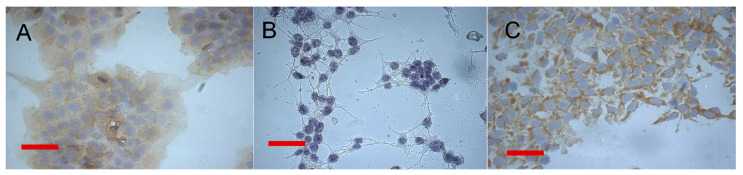
Immunocytochemical detection of epidermal growth factor receptor (EGFR) in fixed cells. EGFR was detected using horseradish peroxidase-conjugated anti-EGFR antibodies. DNA was stained with hematoxylin. (**A**)—A431 cells, (**B**)—M3 cells, (**C**)—M3-EGFR cells. Scale bar represents 100 µm.

Keeping in mind that immunochemistry remains a widespread routine “gold standard” method in clinical practice, we further confirmed the presence of EGFR expression in fixed cells using immunocytochemistry. EGFR-positive A431 was used as a positive control, and original M3 served as a negative control. As anticipated, a signal was detected on A431 and M3-EGFR cells, but not on M3 cells, which indicates specific binding of anti-EGFR antibodies with the developed M3-EGFR cell line (Figure 3). One hundred percent of the viewed M3-EGFR cells stained positive for EGFR.

Semi-quantification of the immunocytochemical data verified that EGFR staining of M3-EGFR cells is significantly more intense than EGFR staining of parental M3 cells (*p* < 0.001) (Figure S1).

### 3.3. Study of MNT Endocytosis in M3-EGFR Cells

**Figure 4:**
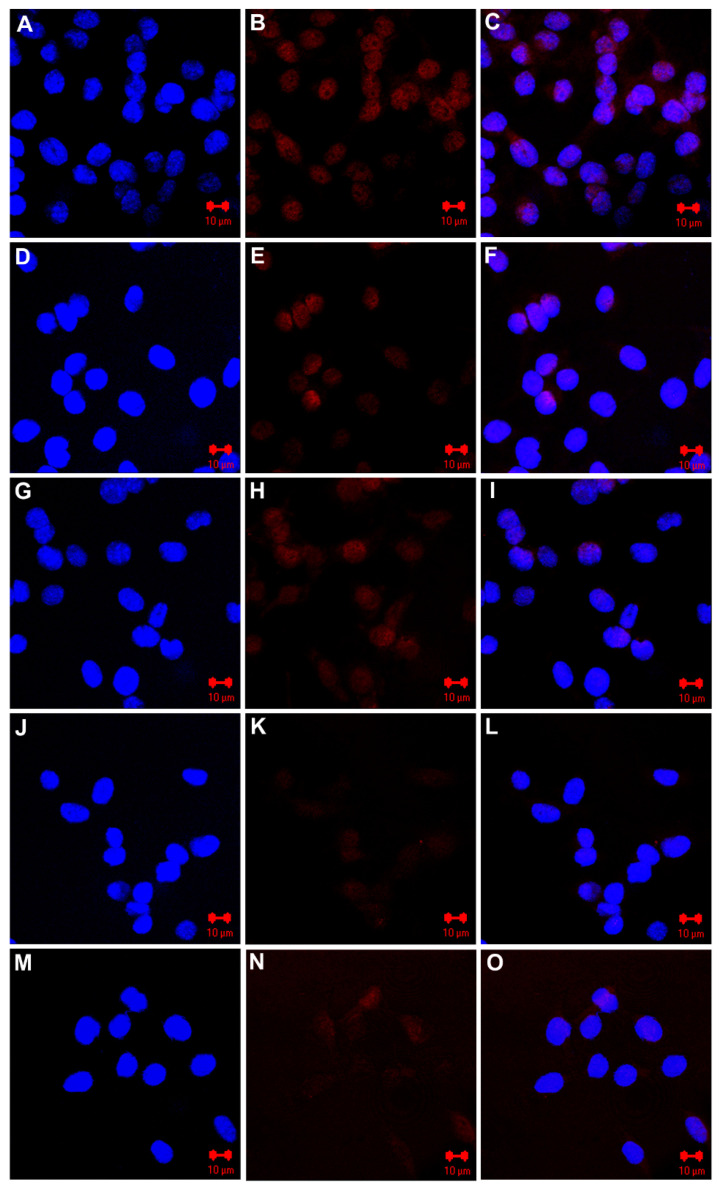
Representative images of fixed M3-EGFR cells obtained by confocal laser scanning microscopy. Incubation with MNT-EGF for 2 h (**A**–**C**), with EGF-MNT (**D**–**F**), affibody-MNT (**G**–**I**), non-targeted MNT (**J**–**L**), and without MNT (**M**–**O**), where (**A**,**D**,**G**,**J**,**M**)—nuclei stained with 4′,6-diamidino-2-phenylindole; (**B**,**E**,**H**,**K**,**N**)—epidermal growth factor receptors revealed by immunofluorescence using Alexa Fluor 647 conjugated antibody; (**C**,**F**,**I**,**L**,**O**)—combined images. Scale bar represents 10 µm.

The ability of EGFR-targeted MNT to enter M3-EGFR cells and undergo subsequent intracellular trafficking to nucleus was evaluated using confocal laser scanning microscopy. Following 2-h incubation with MNT-EGF, Alexa Fluor 647 fluorescence representing MNT distribution was detected in M3-EGFR with predominant localization in the cellular nuclei (Figure 4A–C). Experiments performed with other MNTs further confirmed receptor-mediated endocytosis into M3-EGFR cells and subsequent transport into the cell nuclei (Figure 4D–I). Incubation with non-targeted MNT lacking EGFR-targeting ligand module results in a sharply reduced immunofluorescent signal within the cells (Figure 4J–L) similar to that of control cells incubated without MNT (Figure 4M–O), demonstrating the specificity of the developed M3-EGFR cells to bind and internalize EGFR-targeted molecules.

**Figure 5:**
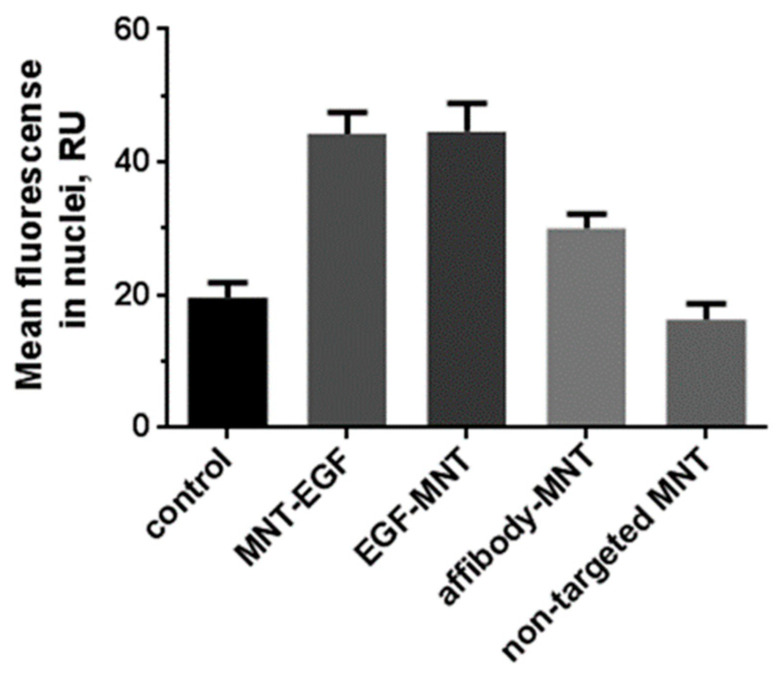
Uptake of various modular nanotransporters (MNTs) by M3-EGFR cells as determined by immunofluorescent analysis. The value of the average fluorescence of the nuclei of M3-EGFR cells fixed after a two-hour incubation with the following MNTs: non-targeted MNT (n = 37), MNT-EGF (n = 118), EGF-MNT (n = 75) and affibody-MNT (n = 79). Data are presented as means ±95% CI. RU—relative units.

Quantitative analysis of the immunofluorescent signal associated with cell nuclei revealed that the MNT-EGF and EGF-MNT constructs proved to be the most effective: the average fluorescence of M3-EGFR cell nuclei after incubation with these MNTs was 44.3 ± 1.3 and 44.7 ± 2.2 Relative units (RU), respectively, and do not differ significantly from each other (*p* = 0.99). The fluorescent signal from the nuclei of cells incubated with the non-targeted MNT did not differ from the signal obtained from M3-EGFR cells incubated without MNT (*p* = 0.759) (Figure 5). The fluorescence of the cell nuclei after incubation with any MNT, except the non-targeted one, differed from the control value significantly (*p* < 0.0001).

### 3.4. Flow Cytometry Studies of Cetuximab-Cy3 Interaction with M3-EGFR Cells

**Figure 6:**
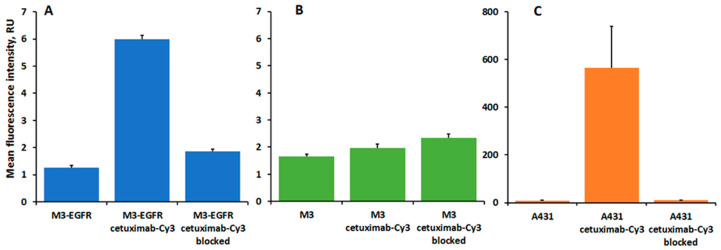
Flow cytometry study measuring interaction of human EGFR specific antibody cetuximab-Cy3 with M3-EGFR (**A**), parental M3 (**B**) and A431 cells (**C**). Cetuximab-Cy3 blocked—refers to cells incubated with Cetuximab-Cy3 in the presence of 1 µM non-labeled cetuximab and 1 µM of human EGF Untreated cells served as autofluorescence controls. Error bars represent standard errors of mean (n = 3–5).

In order to determine whether the EGFR expressed on M3-EGFR cells is of human origin, we used human EGFR-specific antibody cetuximab [25,26,27] to assess its interaction with M3-EGFR cells by flow cytometry. We found that cetuximab-Cy3 bound specifically to M3-EGFR and human EGFR positive A431 cells, while no specific binding was detected for parental M3 cells (Figure 6).

### 3.5. EGFR Expression in M3-EGFR In Vivo

**Figure 7:**
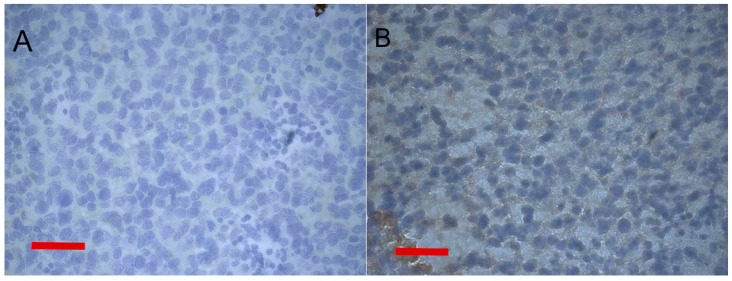
Micrographs of mouse melanoma sections with immunohistochemical verification of epidermal growth factor receptor (EGFR) expression. DNA was stained with hematoxylin. (**A**)—M3 tumor section. (**B**)—M3-EGFR tumor section. Scale bars represent 100 μm.

The developed M3-EGFR cells demonstrated 100% tumorigenicity with tumors developed 2–4 weeks after inoculation to DBA/2 mice (n = 7). The M3-EGFR tumor growth curve was similar to the original M3 one (Figure S2). This made it possible to proceed to the study of human EGFR expression in transplanted subcutaneous tumors of immunocompetent mice. Immunohistochemical staining of tumor sections revealed the presence of specific staining of EGFR on the M3-EGFR mouse tumor, while no staining was seen on the original M3 mouse tumor (Figure 7).

Semi-quantification of the immunohistochemical data verified that EGFR staining of M3-EGFR tumors is significantly more intense than EGFR staining of parental M3 tumors (*p* < 0.03) (Figure S3).

## 4. Discussion

Design of a new anticancer drug is a highly time- and resource-consuming as well as expensive process. Thus, despite its well-known and widely discussed limitations, the syngeneic mouse tumor model remains the cheapest, fastest and most reproducible way to assess anticancer drug efficacy on animals with innate immunity [28].

Based on the literature, there are several tumorigenic mouse cell lines expressing significant amounts of EGFR, which were used for in vivo studies: hepatocellular carcinoma CBO140C12 [29,30], a metastatic variant of Lewis lung carcinoma 3LLD122 [31,32], mouse breast carcinoma 4T1 [33] (ATCC CRL-2539) and mouse colon carcinoma CT26 (ATCC CRL-2638). However, CBO140C12 and 3LLD122 are not deposited in cell depositaries such as ATCC, sharply limiting their availability and quality control. The most widely utilized syngeneic mouse tumor model, CT26, possesses contrary data on its EGFR expression level, varying from undetectable [34] to very high [35]. However, all the aforementioned cell lines are neither of melanoma origin, nor do they express human EGFR, which can be required for testing anticancer agents recognizing specifically human receptors.

More than a decade ago, Diaz et al. published the first record of successful development of a human EGFR-expressing B16-F10 mouse melanoma cell line [15]. The authors verified EGFR expression in selected transfected B16-F10 clones in vitro, then further moved to the in vivo experiments and confirmed the preserved tumorigenicity of the transfected cells in syngeneic C57BL/6 mice. However, the resulting tumors demonstrated a significant and irreversible loss of EGFR in syngeneic mice, while EGFR expression persisted when tumors developed only in immunocompromised animals [16]. In the present study, we developed a Cloudman melanoma S91 clone M3 cell line demonstrating preserved expression of human EGFR both in vitro and, most importantly, in vivo in immunocompetent mice. Unlike the results obtained with EGFR transfected B16-F10 [16], syngeneic M3-EGFR tumors developed in immunocompetent mice retained the receptor expression, according to immunohistochemistry staining. This can be attributed to either the use of another pair of cells and mouse strain, or, more likely, to another transfection method used. We have proven the human origin of EGFR in the developed M3-EGFR melanoma cell line by its ability to bind to human EGFR-specific monoclonal antibodies by flow-cytometry (Figure 6).

The aim of this study was the development of a syngeneic mouse melanoma tumor model for testing anti-cancer agents targeted at human EGFR, and we made progress in this direction. EGFR-targeted modular nanotransporters (MNTs) has been chosen. MNTs are drug delivery systems which exploit cell surface receptor binding and internalization to target cancer cells followed by subsequent transport into the desired cellular compartment, the most vulnerable for the cargo drug. Targeted at various cell surface receptors (including EGFR), several MNTs have already demonstrated their efficacy as an anti-cancer drug delivery system in vitro and in vivo [9,10,36]. We used three different EGFR-targeted MNTs in this study with two different ligand modules (either EGF or EGFR-targeted affibody). The obtained results confirmed the ability of the developed M3-EGFR to bind and internalize specifically and provide the anticipated intracellular nuclear import of all studied MNTs, justifying the functionality of EGFR in the developed cell line.

## 5. Conclusions

As a result, we have successfully developed what is, to the best of our knowledge, the first mouse melanoma model with preserved in vivo expression of human EGFR, suitable for in vivo efficacy studies of EGFR-targeted drugs in syngeneic transplantable melanoma tumor setting.

## Data Availability

Not applicable.

## Supplementary Materials

The following supporting information can be downloaded at: https://www.mdpi.com/article/10.3390/pharmaceutics14112448/s1, Figure S1: Semi-quantification of epidermal growth factor receptor (EGFR) expression in M3-EGFR, parental M3 and A431 cell lines. EGFR was detected by immunocytochemistry in fixed cells using horseradish peroxidase-conjugated anti-EGFR antibodies (see Materials and Methods section in the main text). One-way analysis of variance (ANOVA) with Tukey’s multiple comparisons test was carried out to test for significant differences between the means. Data are presented as means ± standard error of mean (n = 10–12); a.u.—arbitrary units. Figure S2: Tumor growth curves of M3-EGFR and parental M3 tumors in DBA/2 mice after subcutaneous inoculation of 2 million cells. Data are presented as means ± standard error of mean (n = 7–19). Figure S3: Semi-quantification of epidermal growth factor receptor (EGFR) expression in M3-EGFR and parental M3 tumors. EGFR was detected by immunohistochemistry in fixed tissue sections using horseradish peroxidase-conjugated anti-EGFR antibodies (see Materials and Methods section in the main text). Student t-test was carried out to test for significant differences between the means. Data are presented as means ± standard error of mean (n = 10); a.u.—arbitrary units.
