# Supplementary material for: Mouse Syngeneic Melanoma Model with Human Epidermal Growth Factor Receptor Expression
Source: Pharmaceutics. 2022 Nov 12;14(11):2448. doi: 10.3390/pharmaceutics14112448 (PMC9697344; doi:10.3390/pharmaceutics14112448)
Supplement: Supplementary file 1 [file pharmaceutics-14-02448-s001.zip › pharmaceutics-1969103-supplementary.pdf]

Supplementary Materials for the article

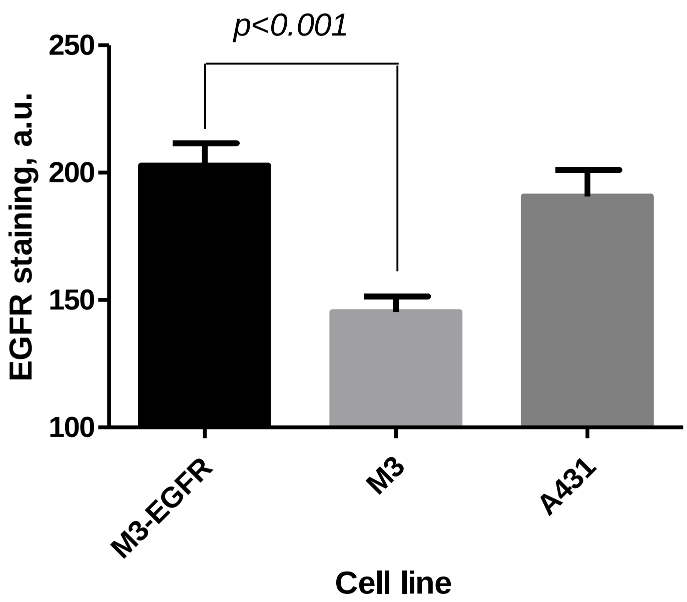

Figure S1. Semi-quantification of epidermal growth factor receptor (EGFR) expression in M3-EGFR, parental M3 and A431 cell lines. EGFR was detected by immunocytochemistry in fixed cells using horseradish peroxidase-conjugated anti-EGFR antibodies (see Materials and Methods section in the main text). One-way analysis of variance (ANOVA) with Tukey's multiple comparisons test was carried out to test for significant differences between the means. Data are presented as means ± standard error of mean (n=10-12); a.u. – arbitrary units.

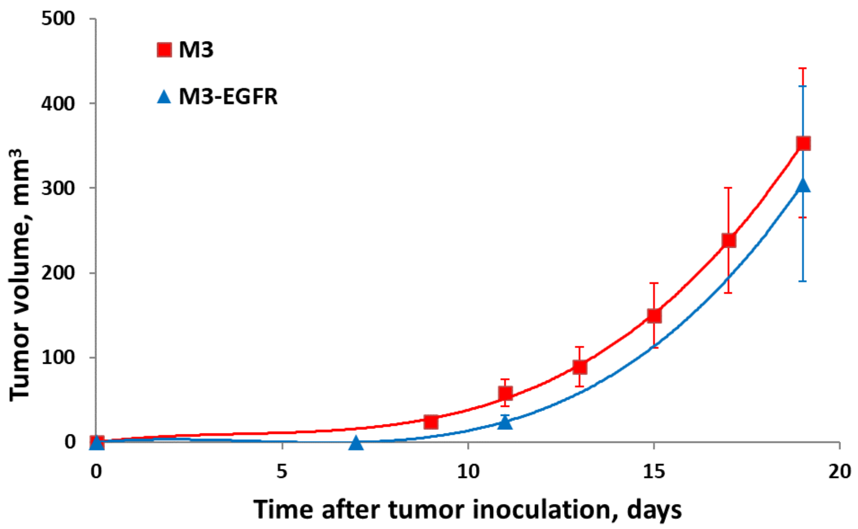

Figure S2. Tumor growth curves of M3-EGFR and parental M3 tumors in DBA/2 mice after subcutaneous inoculation of 2 million cells. Data are presented as means ± standard error of mean (n=7-19)

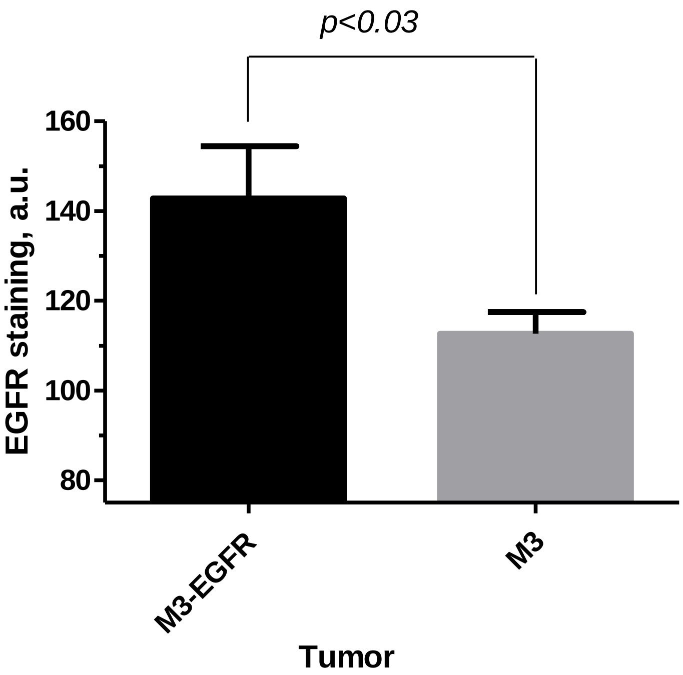

Figure S3. Semi-quantification of epidermal growth factor receptor (EGFR) expression in M3-EGFR and parental M3 tumors. EGFR was detected by immunohistochemistry in fixed tissue sections using horseradish peroxidase-conjugated anti-EGFR antibodies (see Materials and Methods section in the main text). Student t-test was carried out to test for significant differences between the means. Data are presented as means ± standard error of mean (n=10); a.u. – arbitrary units.
